# Enrichment of Denitrifying Methane-Oxidizing Microorganisms Using Up-Flow Continuous Reactors and Batch Cultures

**DOI:** 10.1371/journal.pone.0115823

**Published:** 2014-12-29

**Authors:** Masashi Hatamoto, Masafumi Kimura, Takafumi Sato, Masato Koizumi, Masanobu Takahashi, Shuji Kawakami, Nobuo Araki, Takashi Yamaguchi

**Affiliations:** 1 Department of Civil and Environmental Engineering, Nagaoka University of Technology, Nagaoka, Niigata, 940-2188, Japan; 2 Department of Civil and Environmental Engineering, Tohoku University, Sendai, Miyagi, 980-8579, Japan; 3 Department of Construction Systems Engineering, Anan National College of Technology, Anan, Tokushima, 774-0017, Japan; 4 Department of Civil Engineering, Nagaoka National College of Technology, Nagaoka, Niigata, 940-8532, Japan; Arizona State University, United States of America

## Abstract

Denitrifying anaerobic methane oxidizing (DAMO) microorganisms were enriched from paddy field soils using continuous-flow and batch cultures fed with nitrate or nitrite as a sole electron acceptor. After several months of cultivation, the continuous-flow cultures using nitrite showed remarkable simultaneous methane oxidation and nitrite reduction and DAMO bacteria belonging to phylum NC10 were enriched. A maximum volumetric nitrite consumption rate of 70.4±3.4 mg-N·L^−1^·day^−1^ was achieved with very short hydraulic retention time of 2.1 hour. In the culture, about 68% of total microbial cells were bacteria and no archaeal cells were detected by fluorescence *in* *situ* hybridization. In the nitrate-fed continuous-flow cultures, 58% of total microbial cells were bacteria while archaeal cells accounted for 7% of total cell numbers. Phylogenetic analysis of *pmoA* gene sequence showed that enriched DAMO bacteria in the continuous-flow cultivation had over 98% sequence similarity to DAMO bacteria in the inoculum. In contrast, for batch culture, the enriched *pmoA* gene sequences had 89–91% sequence similarity to DAMO bacteria in the inoculum. These results indicate that electron acceptor and cultivation method strongly affect the microbial community structures of DAMO consortia.

## Introduction

Microbial consortia that perform denitrifying anaerobic methane oxidation (DAMO) have been discovered in the last decade [1], [2]. The microorganisms responsible for the DAMO reaction belong to the previously uncultured NC10 phylum and an archaeal group distantly related to anaerobic methanotrophic (ANME) archaea. Recently, the complete genome of a bacterium in the phylum NC10, named “*Candidatus* Methylomirabilis oxyfera”, was assembled from metagenomic sequencing of an enrichment culture of DAMO [3]. From isotopic labeling experiments and the genomic data, *M. oxyfera* has an intra-aerobic methane oxidation pathway and oxidizes methane using the oxygen produced through the dismutation of nitric oxide to oxygen and nitrogen gas. Using the DAMO reaction, *M. oxyfera* bypasses the production of nitrous oxide, a canonical intermediate of standard denitrification pathways. Very recently, the genome sequence of DAMO archaea, named “*Candidatus* Methanoperedens nitroreducens” was reported, confirming the role of an archaeon in the DAMO reaction [4]. Contrary to *M. oxyfera, M. nitroreducens* oxidizes methane by a reverse methanogenesis pathway and reduces nitrate to nitrite. Microorganisms capable of DAMO have great potential for development of a novel denitrification process that could reduce the production of greenhouse gases nitrous oxide and methane.

Nitrogen removal processes from wastewater using DAMO microorganisms have been investigated recently. Kampman et al. (2012) [5] reported denitrification using dissolved methane from the effluent of a sewage treatment upflow anaerobic sludge bed reactor. Nitrogen removal using coupling of anammox and DAMO microorganisms was also verified, and a coupling process of anammox and DAMO using a membrane biofilm reactor was reported [6]–[8]. These processes could potentially be applicable for nitrogen removal in the near future. However, Kampman et al. (2012) pointed out that an order of magnitude increase of nitrite consumption rate was needed for practical application of DAMO microorganisms for wastewater treatment. In addition, the long doubling times of ∼1–2 weeks [1], [9], negatively influence the feasibility of using DAMO for denitrification processes. Previous studies have typically used DAMO biomass from sequencing batch reactor (SBR) cultures inoculated with sediment samples, peatland soil, and wastewater sludges [10]–[13]. On the other hand, the effects of cultivation method on the enrichment of DAMO microorganisms have not received much attention.

In this study, we used paddy field soil as an inoculum source and enriched DAMO microorganisms using two different cultivation methods, batch cultivation and continuous flow culture. Enriched DAMO consortia were evaluated by fluorescence in-situ hybridization (FISH) analysis and *pmoA* gene-based phylogenetic analysis to reveal the effects of nitrogen source and cultivation method.

## Materials and Methods

### Sampling site

A total of three paddy field soil samples (site KT; 37°25′29″N, 138°47′9″E, site SE; 37°25′19″N, 138°47′12″E, site SZ; 37°25′33″N, 138°47′31″E, Nagaoka, Japan) were collected and analyzed in this study. These paddy field soils were fine gley soil and culturing Koshihikari rice with common fertilization methods of Niigata prefecture. The sites were on private land with permission from the landowner for soil sampling. Samples were collected from 10–20 cm below the soil surface at 10 cm water depth. Surface water temperature and pH readings were 22.5°C, 7.32, 23.2°C, 7.46, and 19.5°C, 7.25 for site KT, SE, and SZ, respectively.

### Enrichment condition

Paddy field soils samples were used as inoculum for continuous cultures and batch cultures. For continuous culture, cylindrical glass column bioreactors (diameter, 5 cm; length, 13 cm) with a coarse sponge sheet as the biofilm carrier material was used. The coarse sponge sheet was soaked in diluted paddy field soil and inoculated the biomass. A synthetic medium composed of the following was used (per liter): KHCO_3_, 500 mg; KH_2_PO_4_, 50 mg; CaCl_2_·2H_2_O, 300 mg; MgSO_4_·7H_2_O, 200 mg; an acidic trace element solution, 0.5 ml; and an alkaline trace element solution, 0.2 ml. The compositions of the acidic trace element solution and the alkaline trace element solution were prepared according to previous reports [11]. Two identical continuous bioreactors were operated with NaNO_2_ (0.5 to 1.0 mM) and NaNO_3_ (0.5 mM) added to reactor A and B, respectively. The medium was flushed with argon and methane, and then the headspace of the bottle was filled with methane and set at an initial flow rate of 64 ml h^−1^, correspondent to a hydraulic retention time (HRT) of 4.2 h. Absence of dissolved oxygen in the medium bottles were checked by a Clark type oxygen electrode. pH of the medium was around 7.4. Cultures were maintained at 30°C.

For batch cultures, 720 ml glass serum bottles were filled with the same medium used in the continuous culture experiments. The liquid volume was 400 ml, and 320 ml headspace of the serum bottle was filled with 100% of methane and closed with butyl stoppers. Cultures were maintained at 30°C in a shaking incubator. To consume the organic matter in the paddy field soil samples, pre-incubation was conducted for 114 days. Methane consumption was observed at the end of the pre-incubation, then the medium was replaced and the batch cultivation experiment was started.

### DNA extraction, PCR, cloning and phylogenetic analysis

DNA was extracted from the washed sludges using the Fast DNA spin kit for soil (MP Biomedicals, Irvine, CA), as described in the manufacturer’s instructions. Extracted DNA was used for amplification of the *pmoA* gene using specific primers of A189b, cmo682, cmo182, and cmo568 (Luesken et al. 2011c). To amplify the *pmoA* gene fragment, primers A189b and cmo682 were used for the first PCR, and cmo182 and cmo568 were used for the second PCR using ONE Shot LA PCR MIX (TAKARA BIO, Otsu, Japan). The conditions for both PCRs were as follows: initial melting step for 5 min at 94°C, followed by 20–35 cycles of denaturation at 94°C for 30 s, annealing at 55°C for 30 s, and elongation at 72°C for 1 min. Finally, an elongation step at 72°C for 10 min was performed. Clone libraries were constructed based on previously described methods [14]. Representative clones having different restriction fragment length polymorphism patterns were then subjected to sequencing.

Sequence data were aligned with the ARB program package [15] and the aligned data were manually checked for chimeras. The phylogenetic trees based on *pmoA* gene sequences were constructed by the neighbor-joining method implemented in the ARB program. Bootstrap resampling analysis for 1,000 replicates was performed to estimate the confidence of tree topologies.

### Oligonucleotide probes, FISH, and cell counting

The 16S rRNA-targeted oligonucleotide probes used in this study were EUB338 [16], EUB338 II, and EUB338 III [17] for total bacteria, NC10-1162 [10], DBACT-193, and DBACT-447 [1] for NC10 bacteria, and ARC915 [18] for archaea. The probes were labeled with Alexa488 or Alexa555. Enrichment samples were fixed with 4% paraformaldehyde in phosphate-buffered saline (137 mM NaCl, 8.1 mM Na_2_HPO_4_, 2.68 mM KCl, 1.47 mM KH_2_PO_4_, pH 7.2), and left for 4 h at 4°C. Hybridization was carried out in hybridization buffer (900 mM NaCl, 20 mM Tris-HCl [pH 7.2], 40% formamide [v/v], 0.01% SDS [w/v], 0.5 µM of fluorescently labeled probe) at 46°C for at least 3 h. After hybridization, the slides were washed at 48°C for 20 min with washing buffer containing the same components as the hybridization buffer except the probes. An Olympus BX53 epifluorescence microscope (Olympus, Tokyo) with color CCD camera VB-7010 (Keyence, Osaka) was used for observation of the samples. For quantitative determination of microbial composition in the DAMO reactor sample, the total number of cells was determined by 4′,6′-diamidino-2-phenylindole (DAPI) direct counting. To determine the percentage of FISH-positive cells, at least 30 representative microscopy images were obtained from each sample.

### Analytical methods

The concentrations of nitrite and nitrate were routinely measured by ion chromatography [19]. Dissolved methane in the influent and effluent was measured by the headspace technique described previously [19]. Dissolved methane concentration was calculated based on Henry’s Law using quantities of equilibrated headspace methane and Bunsen’s coefficient.

### Nucleotide sequence accession numbers

The *pmoA* gene sequence data obtained in this study were deposited in the GenBank/EMBL/DDBJ databases under accession numbers AB767281 to AB767293.

## Results

### Screening of seed sample

**Figure 1:**
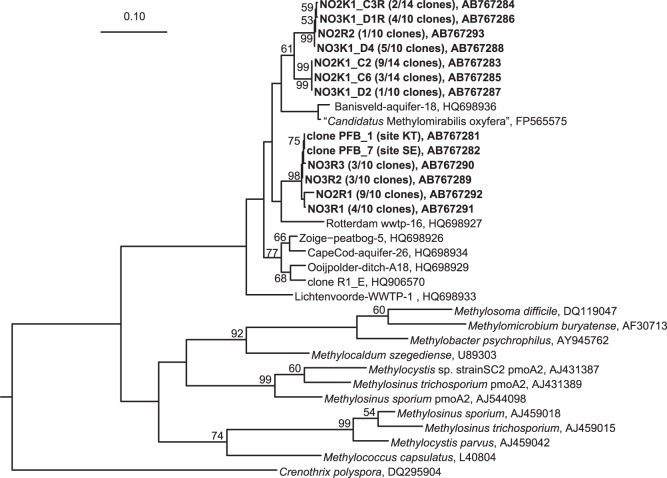
Phylogenetic tree constructed with the neighbor- joining method of the *pmoA* genes identified from paddy field soil and enrichment cultures. The sequence of the *amoA* gene from Nitrosomonas europaea (L08050) was used as an outgroup. Clones obtained in this study are in bold: clones in the series “NO2K-”, “NO3K-” were obtained from the batch cultures A and B, respectively; clones “NO2R-”, “NO3R-” were obtained from the continuous culture reactors A and B, respectively. The scale bar represents the number of nucleotide changes per sequence position. The numbers at each branch point are bootstrap values obtained by 1,000 resampling analysis.

To select an appropriate seeding for enrichment of DAMO bacteria, three paddy field soil samples were screened with the *pmoA* gene targeted PCR and phylogenetic analysis assay. In the previous studies, *M. oxyfera*-related typical DAMO bacteria were detected and enriched from paddy fields soils, sediments of ditches or freshwater, and biomass from a municipal wastewater treatment plant [5], [10]–[12], [20], so the flooded paddy field soils were considered suitable in this study. In two out of three tested paddy field soils (KT and SE), the *M. oxyfera*-related *pmoA* gene could be detected (Fig. 1). Both clones had the same gene sequence, which was 87% and 89% similar to the *pmoA* sequence from “*M. oxyfera*” and its closest clone Rotterdam-WWTP-16, respectively. Thus, these two paddy field soils samples, KT and SE, were used as an inoculum for following the enrichment experiments.

### Enrichment of DAMO microorganisms using continuous reactor

**Figure 2:**
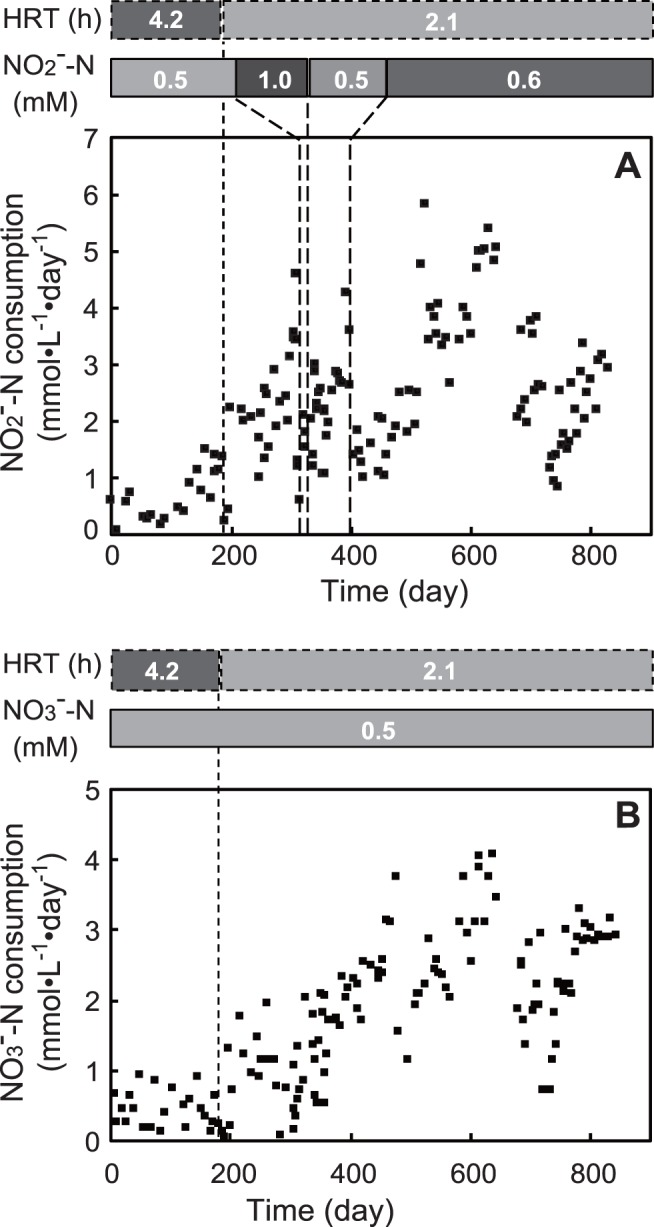
Time course of nitrite (A) and nitrate (B) consumption rate of continuous cultures.

About a 100 days after the bioreactor cultivation was started, nitrite concentrations in the effluent of reactor A decreased compared to the influent concentration of 0.5 mM, and the nitrite consumption rate began to increase, reaching 4.62 mmol·L^−1^·day^−1^ on day 307 with a removal ratio of 78% (Fig. 2). When the influent nitrite concentration was doubled to 1 mM, the nitrite removal ratio and consumption rate dropped sharply. Influent nitrite concentration was set back to the initial concentration of 0.5 mM, but recovering the removal rate took three months. Subsequently, the influent concentration of nitrite was slightly increased to 0.6 mM and the nitrite removal ratio and consumption rate dropped sharply again. After several months of cultivation, the nitrite consumption rate increased and maximal consumption was achieved at 5.03±0.22 mmol·L^−1^·day^−1^ (70.4±3.4 mg-N·L^−1^·day^−1^) day 608 to 643 (Fig. 2). Compared to reactor A, the nitrate fed reactor B had a longer cultivation period to increase the nitrate consumption rate. It required about one year to observe increasing of nitrate consumption and the maximal consumption rate was 3.64±0.42 mmol·L^−1^·day^−1^ (51.0±5.9 mg-N·L^−1^·day^−1^) on day 608 to 643.

Measurements of dissolved methane concentrations in the influent and effluent indicated (Table 1) that both reactors consumed methane stoichiometrically, consistent with the theoretical DAMO reaction (equations 1, 2).

**Table 1:** Ratio of consumed methane and reduced electron acceptors in continuous cultures in operation days of 794 to 832.

Reactor	Electron acceptor	Nitrite•nitrateconsumption rate(mmol/L/day)	Dissolved methaneconsumption rate(mmol/L/day)	Theoretical methaneconsumption rate*(mmol/L/day)
A	nitrite	2.80±0.37	1.40±0.31	1.04
B	nitrate	2.95±0.10	1.71±0.70	1.84

*Theoretical methane consumption rate was calculated based on nitrate or nitrite consumption rate.



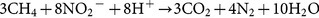
(1)

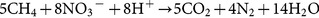
(2)


### Enrichment of DAMO bacteria using batch cultivation

**Figure 3:**
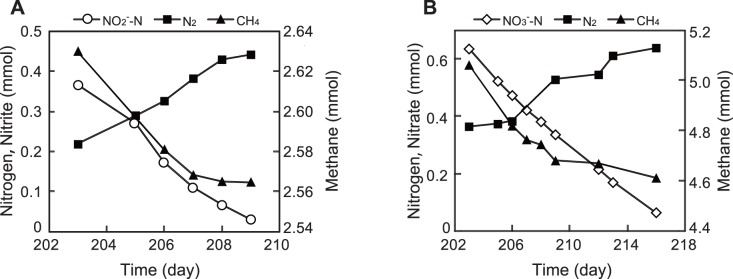
Methane oxidation, denitrification, and nitrite (A) or nitrate (B) reduction of batch cultures.

Fig. 3 shows the consumption of methane, nitrite, and nitrate and production of nitrogen gas after the second medium replacement. The methane and nitrite consumption rates of batch A were 0.03 and 0.13 mmol·L^−1^·day^−1^ respectively when measured over the period of days 203–209 (b 3). The methane consumption rate was close to the theoretical consumption rate of 0.04 mmol·L^−1^·day^−1^, which was calculated based on equation (1) from the nitrite consumption rate. In nitrate fed batch B, the methane and nitrate consumption rates were 0.06 and 0.11 mmol·L^−1^·day^−1^, respectively. The consumption ratio of methane and nitrate was also close to the theoretical ratio calculated based on equation (2).

### *pmoA* gene sequence analysis of enriched microbial communities

To understand the methanotrophic microbial communities established in continuous reactor cultures and batch cultivation, *pmoA* gene sequences were analyzed by cloning. Five phylotypes were detected from the continuous reactor cultures and four out of five phylotypes were very closely related to the phylotype detected in the inoculum samples (Fig. 1). In fact, phylotype NO3R3 from nitrate fed reactor B had the same *pmoA* gene sequence as clones PFB_1 and PFB_7, detected in inoculum samples KT and SE, respectively. From nitrite fed reactor A, nine out of ten clone were grouped into phylotype NO2R1, which had 98% sequence similarity to clones PFB_1 and PFB_7. These clones, enriched in the continuous reactor culture, had 86–87% similarity to *pmoA* from “*M. oxyfera*”.

The *pmoA* gene clones obtained from batch culture represented five phylotypes, two from the nitrite fed batch A and three from the nitrate fed batch B (Fig. 1). The dominant *pmoA* phylotype was different for batch A and batch B. The sequence of the dominant phylotype from batch A (NO2K1_C2) was 93% similar to the dominant phylotype from batch B (NO3K1_D4, NO3K1_D1R). These three phylotypes have 89–91% sequence similarity to the *pmoA* sequence of clones PFB_1 and PFB_7, and 88–90% similarity to the *pmoA* gene of “*M. oxyfera*”.

### Enriched microbial community analysis by FISH

**Figure 4:**
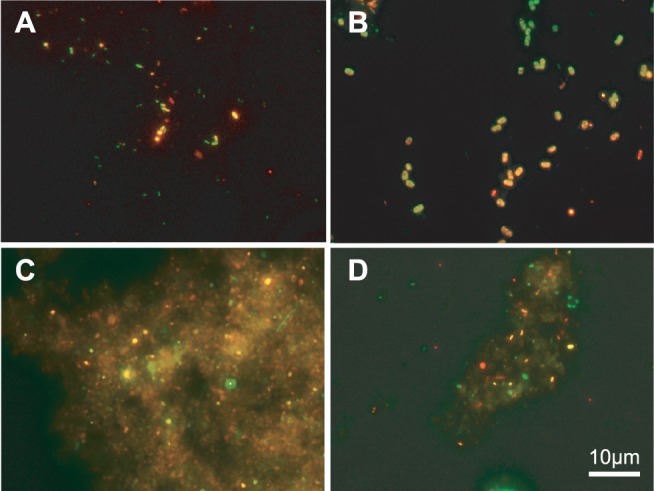
Fluorescence in situ hybridization of biomass from nitrite fed continuous culture (A), nitrate fed continuous culture (B), nitrite batch culture (C), and nitrate batch culture (D). Epifluorescence micrographs taken after hybridization with the general bacterial probes of Alex488-labeled EUB338, EUB338 II, and EUB338 III (green), Cy3-labeled NC10-1162, DBACT-193, and DBACT-447 for NC10 bacteria (Red). Bacteria related to *M. oxyfera* appear yellow due to co- hybridization with both probes.

After 9 months of reactor operation, DAMO bacteria of the NC10 phylum were visibly detected by FISH analysis (Fig. 4). In the nitrite fed reactor A, DABCT-193 and NC10-1162 probes could detect the targeted cells but DBACT-447 positive cells were not observed. On the other hand, in nitrate fed reactor B, DAMO bacteria were detected by DBACT-193 and DBACT-447 probes. In reactor A, 54% of cells were hybridized with NC10 bacteria targeted probes and in reactor B, 37% of cells were hybridized with NC10 bacteria targeted probes. In reactor A, 68% of DAPI stained cells were hybridized with EUB338 probes and archaeal cells were not detected by FISH analysis using the ARC915 probe, but for reactor B, 58% and 7% of cells were detected by EUB338 and ARC915 probes, respectively. These results suggest that the electron acceptor could be affecting the DAMO microbial community structure. Although DAMO bacteria were detected from the batch cultivation by FISH analysis (Fig. 4C, D), cell count was not completed for the severe autofluorescence derived from soil samples.

## Discussion

In this study, NC10 specific primers targeting the *pmoA* gene were used to screen paddy field soils for enrichment of DAMO bacteria. DAMO consortia were successfully constructed by continuous reactor cultivation and batch cultivation. In continuous reactor A, which used nitrite as a sole electron acceptor, nitrite consumption was observed after approximately 100 days. This lag period was relatively short compared to previous reports of three months to over one year [1], [10]–[12]. There was a very sharp decrease in nitrite consumption activity when the concentration of nitrite is increased to 1 mM (Fig. 2A). A toxic effect of nitrite on DAMO consortia is pointed out [21]. Thus in this study, increase of nitrogen loading was achieved by decreasing of HRT. Very recently, the strategy was also reported by Kampman et al. (2014). In this study, the maximum nitrite consumption rate of 5.03±0.22 mmol·L^−1^·day^−1^ (70.4±3.4 mg-N·L^−1^·day^−1^) was achieved with very short HRT of 2.1 h. This rate is faster than previously reported in literature [5], [10], [12], [22]. On the other hand, reactor B did not show an increase in nitrate consumption for nearly one year. In previous studies, denitrification was not observed in nitrate-fed DAMO cultures for more than 200 days [10]. Likewise, nitrate removal was not detected in a nitrate and ammonium fed simultaneous DAMO and anaerobic ammonium oxidation (anammox) reactor for more than 300 days [6]. In our experiments, enrichment of DAMO consortia took longer when nitrate was used as an electron acceptor, which is consistent with these earlier reports. In addition, we observed a nitrite consumption rate that was greater than that of nitrate (Figs. 2, 3), consistent with previous studies [1], [9]–[11]. Analysis of the genome of one of the DAMO bacterium, *M. oxyfera*, suggested an ability to reduce nitrite to nitric oxide and oxidize methane using the oxygen produced through the dismutation of nitric oxide to oxygen and nitrogen gas [3]. Thus nitrate reduction to nitrite is performed by organisms other than *M. oxyfera* and that this reaction is the rate-limiting step in the pathway. A novel member of the ANME archaeal lineage, ANME-2d was previously hypothesized to be responsible for the nitrate reduction in DAMO consortia [1], [10]. Recently, Haroon et al. (2013) revealed the role of the ANME-2d archaea in DAMO consortia and proposed the name “*Candidatus* Methanoperedens nitroreducens”. *M. nitroreducens* oxidizes methane to carbon dioxide using a reverse methanogenesis pathway in which nitrate is reduced to nitrite. Previous reports showed that the growth of ANME archaea was extremely slow [23], which is also the case for *M. nitroreducens*. Thus, further research will be needed to optimize the cultivation of *M. nitroreducens* for the application of DAMO to wastewater treatment.

In contrast to continuous cultivation, the consumption rates of nitrate and nitrite were almost the same for batch cultures in our experiments (Table 1). This suggests either that batch culture is more suitable for the microorganisms responsible for the reduction of nitrate to nitrite or that they could not be retained in the continuous flow reactor due to an extremely slow growth rate. The enriched NC10 bacterial communities were different in the two cultivation methods. In continuous reactor cultivation, the *pmoA* gene sequence of enriched NC10 bacteria was closely related to the sequence detected from the original inoculum, except for clone NO2R2 (Fig. 1). On the other hand, for batch cultivation, two phylotypes were detected, but the sequences were distant from the inoculum sequence (Fig. 1). Cultivation methods can have a strong influence on the cultured microbial composition and previous reports have also indicated that continuous flow cultivation techniques could result in successful culture of fastidious microbes from natural environments [24], [25] as we have seen for DAMO.

In conclusion, DAMO microorganisms were enriched from paddy field soils using batch and continuous flow cultivation methods and nitrate or nitrite as the sole electron acceptor. Both the type of electron acceptor and the culture method affected the enriched microbial community structures including the types of NC10 bacteria observed. We have demonstrated that efficient enrichment of NC10 bacteria from paddy field sediments can be achieved with continuous flow culture. The results will provide profound insights into development and operation of DAMO biofilm reactors.
